# Maternal Obesity and Perinatal Depression: An Updated Literature Review

**DOI:** 10.7759/cureus.10736

**Published:** 2020-09-30

**Authors:** Lauren B Pavlik, Katrina Rosculet

**Affiliations:** 1 Obstetrics and Gynecology, Medical College of Wisconsin-Green Bay, De Pere, USA; 2 Radiology, Medical College of Wisconsin-Green Bay, De Pere, USA

**Keywords:** maternal obesity, obesity, perinatal depression, postpartum depression

## Abstract

The objective of this review was to determine if there is an association between maternal obesity and increased risk of perinatal depression. Original research articles were found by conducting an electronic database search of PubMed, ClinicalKey, PsycINFO, and Cochrane Library. Seven articles, published in the last five years, were reviewed. Of the seven articles, five demonstrated an association between some level of maternal obesity and increased risk of perinatal depressive symptoms. The two remaining articles did initially find an association, but it was no longer significant after adjusting for or mediating the analysis with covariates. There appears to be an association between peripartum depressive symptoms and some level of maternal obesity and its comorbidities. More research is needed to determine the mechanism and degree of the association and its clinical significance.

## Introduction and background

Obesity is a serious medical condition with many comorbidities affecting many aspects of overall health. Obesity is defined as a body mass index (BMI) of 30 kg/m2 or greater, and severe obesity is defined as a BMI of 40 kg/m2 or greater. Unfortunately, obesity rates are increasing in the United States, raising concern as a public health issue. The Centers for Disease Control and Prevention (CDC) reports an obesity prevalence of 42.4% of American adults in 2017-2018 [1]. The prevalence of obesity in women before pregnancy ranged from 16.6% to 27%, as reported by the CDC in 2016. Approximately 50% of women who gave birth in 2014 were either overweight or obese [2]. Obesity in pregnancy is associated with increased risk to the mother and unborn fetus, including increased risk of preeclampsia, gestational diabetes, higher rates of cesarean delivery, post-term delivery, and long-term childhood obesity [3].

Another prevalent health concern in obstetrics and gynecology is major depressive disorder with peripartum onset, commonly known as “postpartum depression”. A recent study by the CDC showed that roughly one in nine mothers will experience peripartum depression [4]. According to the American Psychiatric Association’s Diagnostic and Statistical Manual of Mental Disorders (DSM-5), major depressive disorder is characterized by a period of two weeks or more with five or more of the following symptoms present: depressed mood, anhedonia, appetite changes, change in sleeping pattern, fatigue or low energy, feeling guilty or worthless, difficulty concentrating, psychomotor symptoms, and thoughts of suicide. Either depressed mood or anhedonia must be present. If onset of mood symptoms occurs during pregnancy or within four weeks of delivery, it is considered major depressive disorder with peripartum onset. Research and awareness has largely been on postpartum depression, but, according to the DSM-5, up to 50% of postpartum depressive episodes start during pregnancy [5]. Major depressive disorder with peripartum onset is associated with increased risk of preterm birth, growth restriction, negative effects on neurodevelopment of the fetus, and neurocognitive delay throughout early childhood [6,7]. Recent research has indicated there may be negative health outcomes for the mother and child in association with even subclinical depression symptoms which, do not meet DSM-5 criteria for major depressive disorder [8].

In the general population, obesity is a risk factor for major depressive disorder [9]. There have been numerous studies evaluating the relationship between maternal obesity and risk of peripartum depression, with varying results. Some studies report increased risk of postpartum depressive symptoms in obese populations, whereas some did not [10,11]. A systematic review published in 2015 found that there was in fact an association between maternal obesity and increased risk of peripartum depressive symptoms [12]. However, another literature review published in 2015 regarding the risks associated with obesity in pregnancy did not include the increased risk of peripartum depression [3]. Various mechanisms for the development of postpartum depression have been considered, including alteration of the hypothalamic-pituitary-adrenal (HPA) axis, inflammation, oxidative stress, diet, microbiome, and body image [13-18].

Given the history of mixed results regarding an association between maternal obesity and peripartum depression, the aim of this study is to review the recent literature in order to better describe the relationship and mechanism not only between maternal obesity and postpartum depression, but also between maternal obesity and antenatal depression.

The terms “peripartum depression” and “perinatal depression” are used interchangeably in this review to describe depressive symptoms during pregnancy and/or in the postpartum period. Prenatal or antenatal depression is used when describing depressive symptoms during pregnancy. Postpartum depression is used when describing depressive symptoms only after delivery.

## Review

Methods

An electronic database search was conducted using PubMed, Clinical Key, PsycINFO, and Cochrane Library. The following search phrases were used to find relevant articles: “Maternal obesity AND perinatal depression,” “Maternal obesity AND peripartum depression,” “Maternal obesity AND perinatal depressive symptoms,” and “Maternal obesity AND postpartum depression.”

**Figure 1:**
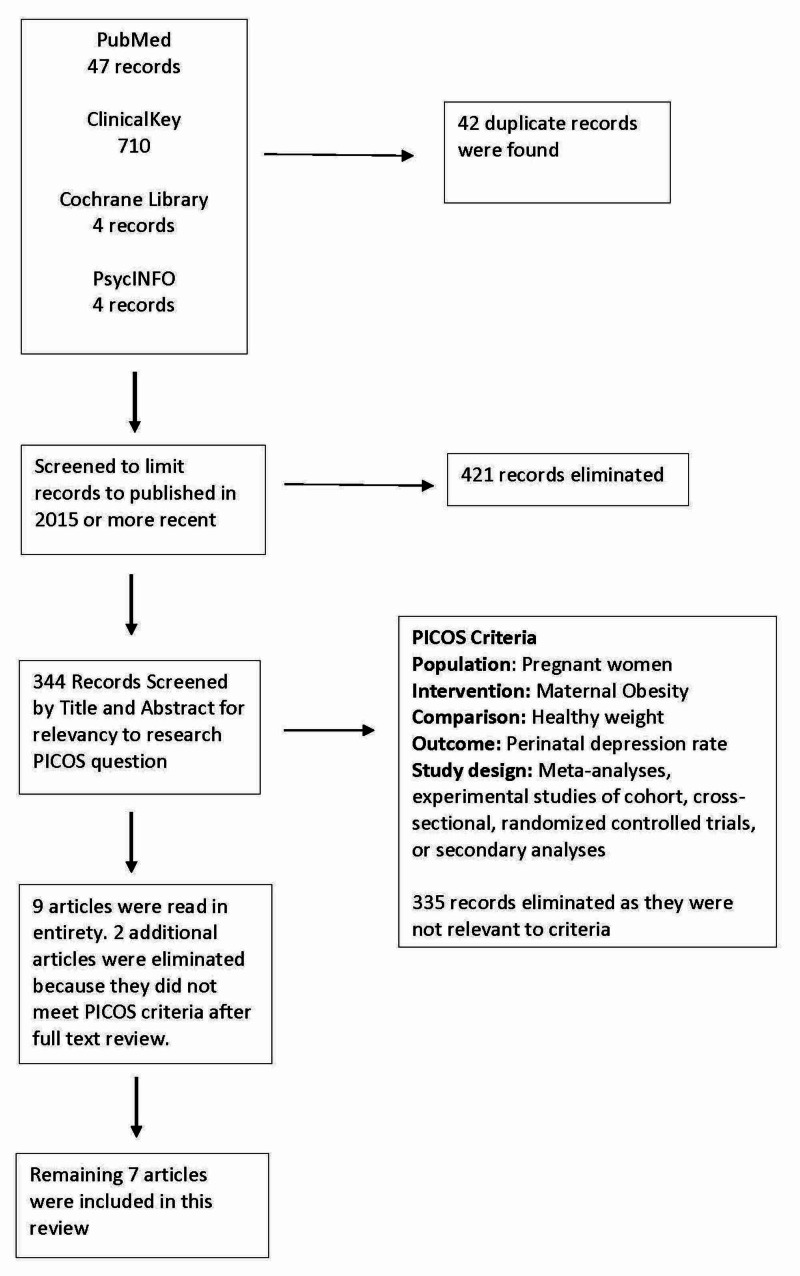
Flowchart of article identification and selection PICOS, Population, Intervention, Comparison, Outcome, and Study Design

A PubMed search of “Maternal obesity AND perinatal depression,” “Maternal obesity AND perinatal depressive symptoms,” and “Maternal obesity AND peripartum depression,” yielded 47 initial articles. A ClinicalKey search of “Maternal obesity AND perinatal depression” yielded 567 journal results initially. An additional search of “Maternal obesity AND peripartum depression” yielded an additional 143 results. A Cochrane Library search of “Maternal obesity AND perinatal depression” yielded four results. PsycINFO search of “Maternal obesity AND perinatal depression” and “Obesity AND perinatal depressive symptoms” yielded four results. Criteria for inclusion in this review are outlined below and demonstrated in Figure 1.

The aim of this review was to evaluate whether there is an association between maternal obesity and risk of perinatal depressive symptoms shown in recent evidence-based research. Due to the difficulty of obtaining relevant randomized controlled trials given the nature of the research question, preferred study designs included prospective, retrospective, and cross-sectional cohort studies. Secondary analyses of randomized controlled trials and cohort studies were also included. Systematic reviews and narrative reviews without any original data or meta-analysis were excluded. As a recent meta-analysis and systematic review was completed in 2015, only articles published in the last five years were selected [3,12]. Duplicate articles and articles not available in English were eliminated. Abstracts were read to evaluate if the article was relevant to the research question. The Population, Intervention, Comparison, Outcome, and Study Design (PICOS) framework used to evaluate relevancy to the research question is included in Figure 1. Articles were excluded if they did not report depression outcomes of obese women compared to normal weight women, as defined by each article’s author. Exclusion criteria were determined before this review was started. Finally, seven original research papers were included in this review. Each article was read in its entirety. Study design characteristics and data were extracted from each article and compiled by the writer.

Results

Description of Reviewed Articles

Characteristics of the seven studies included in the review are presented in Table 1. Of the seven articles reviewed, three were primary analyses and four were secondary analyses. Two of the primary analyses studies were prospective cohort studies, and one study was a retrospective cross-sectional cohort study [19-21]. Of the secondary analyses, three were analyzing prospective cohort studies, and one was an analysis of a randomized controlled trial [22-25]. Four of the studies were based out of the United States, although one study based out of the United States used a Norwegian dataset [19,23-25]. The other three articles were based out of Finland, Australia, and the United Kingdom [20-22]. The average size of study participants was 9,094 women, ranging from 357 to 39,915 women.

**Table 1:** Description of reviewed articles BMI, body mass index; OB, obstetrics

Title	Authors	Year Published	Type of Study	Country of Study Origin	Number of Participants Analyzed (N)	Mode and Timing of Maternal BMI Reported	Scales/Metrics Used	When were Depressive Symptoms Measured	% of Obese in Trial
The Association Between Pre-Pregnancy Body Mass Index, Perinatal Depression and Maternal Vitamin D Status: Findings from an Australian Cohort Study	Jani et al. [21]	2020	Retrospective cross-sectional cohort study	Australia	16,528	Measured height and weight recorded at first OB visit (12-14 weeks’ gestation)	Edinburgh Postnatal Depression Scale	Mid pregnancy (12-14 weeks’ gestation)	19.8% (3,274)
Maternal Early Pregnancy Obesity and Depressive Symptoms during and after Pregnancy	Kumpulainen et al. [22]	2018	Secondary analysis of prospective cohort study	Finland	3,234	Measured height and weight recorded at first OB visit (7-10 weeks) (average 8 weeks 4 days’ gestation)	Center for Epidemiological Studies Depression Scale	Biweekly starting 12+0 to 13+6 gestation until delivery AND at 2.4 and/or 28.2 weeks after pregnancy	13% (430)
Postpartum Depressive Symptoms: Gestational Weight Gain as a Risk Factor for Adolescents Who Are Overweight or Obese	Cunningham et al. [25]	2018	Prospective secondary analysis of a randomized controlled trial	USA	505	Self-reported pre-pregnancy height and weight	Center for Epidemiologic Studies Depression Scale	Second trimester, third trimester, and 6 and 12 months postpartum	17.8% (90)
Perinatal Weight and Risk of Prenatal and Postpartum Depressive Symptoms	Ertel et al. [19]	2017	Prospective cohort study	USA	Pregnant: 2,112; postpartum: 1,686	Self-reported pre-pregnancy height and weight	Edinburgh Postnatal Depression Scale	Mid pregnancy (median: 27.8 weeks’ gestation) and 6 months postpartum	16% (331)
Obesity and the Association with Maternal Mental Health Symptoms	Ruhstaller et al. [23]	2017	Secondary analysis of prospective cohort study	USA	1,010	Self-reported height, weight measured at their first prenatal visit (16-20 weeks)	Center for Epidemiologic Studies Depression scale	Mid pregnancy (16-20 weeks’ gestation)	35% obese (355), Further classified: Class 1: 159 Class II: 102 Class III: 94
Body Image Mediates the Depressive Effects of Weight Gain in New Mothers, Particularly for Women Already Obese: Evidence from the Norwegian Mother and Child Cohort Study	Han et al. [24]	2016	Secondary analysis of a prospective cohort study	USA	39,915	Self-reported pre-pregnancy height and weight	Hopkins Symptom Checklist (SCL-8)	17 weeks’ gestation (time 0), and 18 months (time 1) and 36 months (time 2) postpartum	9.3% (3,731)
Associations of Mood Symptoms with Ante- and Postnatal Weight Change in Obese Pregnancy Are Not Mediated by Cortisol	Mina et al. [20]	2015	Prospective cohort study	United Kingdom	357	Weighed at weeks 17, 28, and 36 of pregnancy and 3 months postpartum	Psychosocial risk factor assessment, satisfaction with Life Scale, General Health Questionnaire, Hospital Anxiety and Depression Scale, and State-Trait Anxiety Index	17 weeks’ gestation, 28 weeks’ gestation, and at the postpartum visit	62% (222)

Maternal obesity was presented as BMI, and all studies used the World Health Organization (WHO) categories defined as underweight (BMI < 18.5 kg/m^2^), normal weight (BMI 18.5-24.9 kg/m^2^), overweight (BMI 25-29.9 kg/m^2^), and obese (BMI ≥ 30 kg/m^2^). Two studies further classified obesity according to the WHO guidelines as follows: class I (BMI 30-34.9 kg/m^2^), class II (BMI 35-39.9 kg/m^2^), and class III (BMI ≥ 40 kg/m^2^) [20,23]. Of note, one study included only obese mothers who were class III obesity or higher [20].

Timing and method of determining maternal BMI varied across the studies. Three of the studies calculated maternal BMI based on self-reported pre-pregnancy height and weight [19,24,25]. Ruhstaller et al. used self-reported height but measured weight at the women’s first prenatal visit, ranging from 16 to 20 weeks’ gestation [23]. Jani et al. measured height and weight at the first prenatal visit, ranging from 12 to 14 weeks’ gestation [21]. Kumpulainen et al. measured height and weight at the first prenatal visit, ranging from 7 to 10 weeks’ gestation [22]. Finally, Mina et al. measured height and weight at 17 weeks’ gestation [20].

Various scales or metrics were used to assess peripartum depressive symptoms. Three studies used the Center for Epidemiological Studies Depression Scale (CES-D) [22,23,25]. Two studies used the Edinburgh Postnatal Depression Scale (EPDS) [19,21]. One study used the Hopkins Symptom Checklist (SCL-8) [24]. Finally, one study assessed mood symptoms including depressive and anxiety symptoms, which were compiled from a combination of the following five tools: Psychosocial Risk Factor Assessment, Satisfaction with Life Scale, General Health Questionnaire, Hospital Anxiety and Depression Scale, and State-Trait Anxiety Index [20].

Timing of the depressive symptom measurement varied. Two studies measured only prenatal depressive symptoms mid-pregnancy [21,23]. The remaining studies measured prenatal and postpartum depressive symptoms [19,20,22,24,25]. The timing of the postpartum measurement ranged from 2.4 weeks postpartum to 36 months postpartum. Exact timing of each study is outlined in Table 1.

Prenatal and Postpartum Depressive Symptom Outcomes

A summary of the results of each study is summarized in Table 2. Of the seven reviewed articles, five studies show an association between some level of pre-pregnancy obesity and either prenatal or postpartum depressive symptoms even after adjusting for common covariates [19,20,22-24]. The remaining two studies did not find a statistically significant association after adjusting for or mediating with other variables [21,25].

**Table 2:** Summary of study results BMI, body mass index; CI, confidence interval; OR, odds ratio; REAP, rapid eating assessment for patients, a 10-item scale that measures nutritional habits; WAVE, weight, activity, variety, excess, a four-item scale that measures activity

First Author, Year	N	Major Results	Covariates	Conclusions of Author
Jani et al., 2020 [21]	16,528	Obese early-pregnancy BMI was associated with increased odds of perinatal depression with an OR of 1.421 (95% CI: 1.91-1.696; P<0.001). However, mediational analysis revealed the association between high BMI and prenatal depression risk was mediated by vitamin D levels.	Country of birth, marital status, mother's working status, smoking, domestic violence, hypertension during pregnancy, season of birth, gender of neonate, maternal age at delivery time, infant birth weight, and parity (if significant at P<0.1)	Obesity did not have a significant association with prenatal depression risk after including vitamin D as a mediator
Kumpulainen et al., 2018 [22]	3,234	Maternal obesity was associated with a 1.43-fold increase of odds of having depressive symptoms throughout pregnancy (95% CI: 1.15-1.77; p<0.001) and a 1.36-fold increase in odds of depressive symptoms postpartum (95% CI: 1.07-1.71; p=0.01) even when women who reported depressive symptoms during pregnancy were excluded.	Maternal age at delivery, smoking during pregnancy, parity, child's gestational age, birth weight, sex, maternal alcohol use during pregnancy, maternal leisure-time physical activity during pregnancy, education level, and history of depression	Maternal obesity is associated with higher rates of depressive throughout pregnancy and postpartum
Cunningham et al., 2018 [25]	505	Obese adolescent mothers who gained excessive gestational weight had significantly increased postpartum depressive symptoms compared to normal weight adolescent mothers who also gained excessive gestational weight. Adolescent mothers who gained recommended gestational weight were not at increased risk of depressive symptoms, regardless of pre-pregnancy BMI.	Maternal age, race or ethnicity, relationship status, postpartum weight retention, dietary habits using the modified version of REAP, and physical activity using WAVE, group vs individual care	Excessive gestational weight gain is an independent predictor of greater postpartum depressive symptoms in obese adolescent mothers. Pre-pregnancy obesity with normal gestational weight gain was not associated with increased postpartum depressive symptoms.
Ertel et al., 2017 [19]	2,112	Pre-pregnancy obesity was associated with higher odds of postpartum depressive symptoms (OR: 1.69; 95% CI: 1.01-2.83). Additionally, obese women who retained 5 kg or more than their pre-pregnancy weight have further increased risk with an OR of 2.23 and 95% CI of 1.09-4.55. This data did not show a significant association with pre-pregnancy obesity and prenatal depressive symptoms.	Age, race/ethnicity, nativity, education, marital status, household income, parity, pregnancy intention, history of depression, and smoking	Obesity does have a significant association with postpartum depressive symptoms, especially in obese women who retain more than 5 kg postpartum. Additional prevention and screening for postpartum depression in obese mothers may be indicated.
Ruhstaller et al., 2017 [23]	1,010	Obesity was not significantly associated with a score consistent with major depressive disorder after adjusting for nulliparity, African-American race, Medicaid insurance, chronic hypertension, and pregestational diabetes. However, class III obesity mothers had symptoms of major depression 3.27 times more than normal weight mothers.	Nulliparity, African-American race, Medicaid insurance, chronic hypertension, and pregestational diabetes	Morbidly obese mothers had increased risk of depressive symptoms. Additional prevention and screening for perinatal depression may be warranted for morbidly obese mothers.
Han et al., 2016 [24]	39,915	Compared to mothers within normal range BMI, mothers with a higher BMI have an increased risk of depressive symptoms 18 months postpartum and tended to have a poorer body image. Positive body image is not protective against depressive symptoms in obese mothers, as compared to overweight and normal weight women. Roughly 12% of depressive symptoms 18 months postpartum are mediated by negative body image.	Level of education, parental income and immigration, and health status	Obesity is associated with poor body image and increased risk of depressive symptoms 18 months postpartum. Poor body image is also associated with increase rate of depressive symptoms in overweight and normal weight women.
Mina et al., 2015 [20]	357	Severe pre-pregnancy maternal obesity was associated with increased anxiety and depressive symptoms throughout pregnancy. Depressive symptoms were associated with excessive gestational weight gain in both normal weight and severely obese mothers, independent of circulating glucocorticoids.	Traumatic obstetric history, reproductive problems, inflammatory disorders, pregnancy complications, sleep-disordered breathing, and history of mental illness	Cortisol does not mediate the higher antenatal and postpartum anxiety and depressive symptoms in severely obese mothers.

The study by Kumpulainen et al. showed a significant association between all severities of maternal obesity and increased risk of both prenatal and postpartum depressive symptoms [22]. Mina et al. showed a significant association between Class III obesity and increased prenatal and postpartum depressive symptoms, but they did not evaluate class I and class II obesity [20]. Ruhstaller et al. showed a significant association between only class III obesity and increased prenatal depressive symptoms after adjusting for covariates, but they did not evaluate postpartum depressive symptoms [23]. Jani et al. showed a statistically significant association between obesity and increased prenatal depressive symptoms, but it was not statistically significant after including mediation analysis of vitamin D levels [21]. Ertel et al. showed a significant association between all classes of obesity and postpartum depressive symptoms, but they did not find a statistically significant association between obesity and prenatal depressive symptoms [19]. Han et al. showed a statistically significant association between obesity and increase in postpartum depressive symptoms but did not evaluate prenatal depressive symptoms [24]. Finally, Cunningham et al. did not find a statistically significant association between obesity and postpartum depressive symptoms independent of gestational weight gain [25].

Of those studies that found an association between maternal obesity and perinatal depressive symptoms, additional findings were also reported. Ertel et al. reported an association between maternal BMI and postpartum depressive symptoms when adjusting for those with a history of depression [19]. This was also shown by Kumpulainen et al. who reported increased depressive symptoms in obese women even adjusting for history of depression and prenatal depressive symptoms [22]. Mina et al. also found that severely obese women had increased depressive symptoms throughout pregnancy even after excluding those with a history of depression [20].

Ertel et al. also found that excessive gestational weight gain was not associated with prenatal depressive symptoms. The research did show, however, that postpartum weight retention had an association with increased postpartum depressive symptoms in the obese mothers. Specifically, obese women who retain 5 kg or more have an increased risk of postpartum depressive symptoms when compared to non-obese women who also retain 5 kg or more (odds ratio: 2.23; 95% CI: 1.09-4.55) [19]. Han et al., however, reported that gestational weight gain did have an association with postpartum depressive symptoms but in only the obese population. The results showed a 10% increase in BMI in obese mothers increased the risk of developing postpartum depressive symptoms by 18%. This association was not shown in normal weight or overweight mothers [24]. However, Mina et al. reported an association between depressive symptoms and excessive gestational weight gain in both non-obese and morbidly obese mothers [20]. Cunningham et al. found that adolescent mothers who were overweight or obese and gained excessive gestational weight had a significant increase in postpartum depressive symptoms when compared to adolescent mothers who were of normal weight and also gained excessive weight. The mothers who were of normal weight and gained excessive gestational weight did not have an increased risk of depressive symptoms. However, the researchers found that excessive gestational weight gain was independent of pre-pregnancy BMI. Overweight or obese adolescent mothers who gained an appropriate amount of weight during pregnancy were not at increased risk for depressive symptoms. The researchers also reported that regardless of BMI, mothers who had higher levels of physical activity had significantly less depressive symptoms [25].

Associations between depressive symptoms and weight were seen in additional BMI categories. Kumpulainen et al. reported a statistically significant 1.68-fold increase in postpartum depressive symptoms in underweight mothers (95% CI: 1.13-2.53; p=0.01). Underweight women had no significant increase in prenatal depressive symptoms [22]. Jani et al. also reported an increased risk of perinatal depression in underweight mothers [21]. Kumpulainen et al. also found that overweight mothers who were not obese had an increased risk of depressive symptoms throughout pregnancy and in the postpartum period. The odds were found to be 23% higher in the overweight cohort and 43% higher in the obese cohort compared to women whose BMIs are within the normal range [22]. This is in contrast to the results of Ruhstaller et al. who found an association only with women with a BMI greater than or equal to 40 kg/m^2^ [23].

A few possible mechanisms behind increased perinatal depressive symptoms associated with increased body mass were explored. Han et al. reported that both overweight and obese women had a poorer body image than those women within normal BMI range. The researchers also report that a positive body image has a negative association with postpartum depressive symptoms in normal weight women, but that protective effect is not observed in obese mothers. After mediation analysis with bootstrapping, Han et al. reported that 12% of the total impact of BMI on the risk of postpartum depressive symptoms was explained by a negative body image [24]. Mina et al. found that severely obese women had significantly increased anxiety and depressive symptoms in the perinatal period, which were independent of circulating glucocorticoids [20]. Jani et al. evaluated the role of vitamin D deficiency and found that the statistically significant association between maternal obesity and increased perinatal depressive risk was no longer statistically significant with the inclusion of vitamin D as a mediator [21].

Discussion

This study evaluates the association between maternal obesity and perinatal depressive symptoms. Overall, five of the seven studies found an increased risk of either antenatal or postnatal depressive symptoms with some level of obesity after adjusting for several common covariates [19,20,22-24]. The remaining two studies found an association initially, but it was no longer statistically significant after adjusting for additional variables [21,25]. One of these studies that did not find statistical significance had a very different patient population as compared to the other studies [25]. The other study that did not find significance after adjustment of additional variables proposed that variable as the mechanism behind the association of obesity and perinatal depressive symptoms [21]. Both studies and their implications are investigated more fully below. Overall, these results suggest that maternal obesity and its comorbidities do have an association with the development of perinatal depressive symptoms.

Although it is known that obesity is a risk factor for major depressive disorder, three of the studies adjusted for history of depression and still found an additional association with maternal obesity, and one of the studies adjusted for women who had antenatal depressive symptoms with similar results [19,20,22]. These data suggest that obese mothers are at a higher risk for depressive symptoms specific to pregnancy and not simply that these studies are only demonstrating the increased risk of major depressive disorder in obese women.

There were mixed results regarding the effect of excessive gestational weight gain in both obese mothers and the controls. Han et al. reported an association between a 10% increase of BMI of the obese mothers and increased risk of depressive symptoms. However, a 10% increase of an obese BMI is more actual kilograms gained than a 10% increase of a normal weight BMI, which may contribute to the results [24]. Perhaps, the disparity between excessive gestational weight gain and depressive symptoms is explained by the severity of the weight gain. Further studies are needed to evaluate if depressive symptoms are associated with the degree of excessive weight gain in both obese and normal weight mothers. Another explanation for the disparity between studies is the varied population of mothers. The one study that found excessive gestational weight gain to be independent of BMI was a cohort of adolescent obese mothers [25]. This could be representing the strong association between weight and risk of depression in adolescent females [26]. It does not explain, however, why the obese mothers who had an appropriate gestational weight gain were not at increased risk of depressive symptoms. Further studies are needed to evaluate the role of gestational weight gain in obese pregnant adolescents.

The results of three of the studies suggest that depressive symptoms are associated not only with obesity but also with any weight that is outside of the healthy BMI. Kumpulainen et al. found that any BMI outside of the healthy range had an increased risk of postpartum depression [22]. This was supported by Jani et al., who demonstrated an association between underweight mothers and depressive symptoms [21]. These two studies suggest that negative health outcomes are associated with overweight and underweight mothers as well and are not limited to the most obese populations. It is not clear if the underlying etiology behind the mothers’ underweight body mass was adjusted for in these models. History of eating disorders, drug abuse, chronic inflammatory diseases, insufficient food access, and cancer could be included as potential confounders in a future study evaluating depressive risk in underweight mothers. These data are in contrast to the results of Ruhstaller et al., which show an association with depressive symptoms only in the most obese subcategory of obesity [23]. These opposing results are potentially explained by the different confounding factors for which the association was adjusted, specifically, African-American race, Medicaid insurance, chronic hypertension, and pregestational diabetes. Additional studies are needed to further evaluate the depression risk in all BMIs outside of the normal, in addition to the subclasses of obesity.

Regarding the mechanism behind the association, effects of cortisol, body image, and vitamin D were evaluated. Regarding the HPA axis, Mina et al. found that there was not a significant association between morning cortisol levels in mothers and an increased risk of postpartum depression [20]. This result is pertinent as other evidence supports cortisol dysregulation in major depressive disorder. Previous data show that elevated cortisol secretion was associated with melancholic or typical major depressive disorder. Reduced levels of cortisol secretion were associated with atypical major depressive disorder [13]. This negative finding in Mina et al.’s study is significant because it may suggest that the mechanism behind perinatal depression is different than the mechanism of major depressive disorder. Another explanation could be due to the timing of the cortisol measurements. Mina et al. tested morning cortisol, when the body’s natural rhythm of cortisol levels are at their highest [20,27]. Perhaps, the normal surge of cortisol experienced in the morning masks any significant difference between groups. A significant association was found, however, with body image and weight in relation to depressive symptoms, even adjusting for other covariates associated with perinatal depressive symptoms, such as less exercise and worse health status. Interestingly, a positive body image was protective against the development of depressive symptoms in normal weight and overweight mothers but not in obese mothers, suggesting that there are additional factors in the obese population that increase the risk of perinatal depressive symptoms [24]. Regardless, given the body image mediation of BMI and depression risk, future studies are needed to evaluate potential interventions to decrease the risk of depression by improving body image in normal weight, overweight, and obese mothers independent of weight loss. In another study, low levels of vitamin D were found to be mediating the association between obesity and depressive symptoms. High pre-pregnancy weights were associated with both low serum vitamin D levels and increased risk of perinatal depressive symptoms. The study proposes the mechanism in which vitamin D acts as a neuro-steroid hormone to promote brain homeostasis. The impact of maternal sunlight exposure or artificial ultraviolet light exposure independent from serum vitamin D levels was not evaluated [21]. There is some speculation in the literature regarding the accuracy of serum vitamin D levels representing total body stores due to fat sequestration [28]. However, these results warrant further trials evaluating interventional vitamin D supplementation in obese mothers and the rates of perinatal depression. The mechanism behind the association between maternal obesity and perinatal depressive symptoms is not fully understood but is complex and multi-factorial. Both negative body image and low serum vitamin D levels appear to impact the risk of perinatal depressive symptoms.

There were a number of factors that made comparing and drawing conclusions from these seven articles challenging. One factor that may explain the differing results is that four of the seven articles used measured weight from the first prenatal visit ranging from 7 to 20 weeks’ gestation [20-23]. The authors of the articles reported a negligible effect of gestational weight gain impacting the baseline weight measurement. However, there is potential that some patients were not technically obese before becoming pregnant, which could have underestimated the effect of true pre-pregnancy obesity. Although this likely would be of small clinical significance, this could have affected the statistical analysis, particularly in the studies that used baseline weight measured later in pregnancy. On the other hand, the remaining three articles used self-reported pre-pregnancy height and weight, which is susceptible to self-reporting bias and underreporting of obese weights [19,24,25].

The use of various depression scales made comparing results challenging. When comparing results from four different tools, grouping positive screens and negative screens from different scales may result in error. Additionally, when comparing the studies, the depression screenings were performed at differing times during gestation and varying lengths of time postpartum. This could be a source of discrepancy that caused the contrasting results. For example, it is not clear why antenatal and postpartum results differed between the studies. This could suggest that there is a difference between antenatal and postpartum depression and the way it manifests itself on routine depression screens. Finally, the tools use depressive symptoms to stratify probable depression or risk of depression but do not represent a diagnosis of clinical depression. Overall, both the CES-D and EPDS are regarded as adequate screening tools for postpartum depression in obstetric literature, but we cannot assume that all of the positive depression screens in these studies resulted in clinical depression diagnoses [29]. That being said, as mentioned in the introduction, there are data suggesting that even depressive symptoms that do not meet clinical depression criteria have negative health effects on both the mother and baby, suggesting clinical significance in all positive perinatal depression screens [8].

Regarding this literature review, these results are limited as it includes no statistical meta-analysis. Additionally, despite the associations mentioned in the review, given the nature of the cohort studies analyzed, neither causation nor directionality of the associations can be determined. Finally, given the nature of the research question, blinded randomized controlled trials were not obtainable.

## Conclusions

In conclusion, there appears to be an association between peripartum depressive symptoms and some level of maternal obesity. Though the mechanism behind this association remains unclear, both negative body image and low serum vitamin D levels play a role. This association of peripartum depressive symptoms and maternal obesity appears to be independent of the increased lifelong risk of major depressive disorder in obese women. The role of excessive gestational weight gain in obese mothers and the risk of peripartum depressive symptoms is less clear and warrants further investigation. Additionally, further research is needed on the impact of any BMI outside of the normal range, including underweight BMI and extreme morbidly obese BMI, on peripartum depressive symptom risk. Finally, further research is needed to evaluate potential interventions related to body image and vitamin D supplementation to prevent or reduce the risk of peripartum depression in obese women.

In addition to the research questions mentioned above, other future study topics include: Is obesity independent from nutrition in the association with peripartum depression? Related to that question, does the microbiome of obese mothers mediate the relationship with peripartum depression? And, finally, does maternal obesity predict the severity of peripartum depression? More research is needed in this area to further evaluate the degree of this association and the subsequent clinical implications.
